# Correlates of physical activity counseling provided by physicians: A cross-sectional study in Eastern Province, Saudi Arabia

**DOI:** 10.1371/journal.pone.0220396

**Published:** 2019-07-25

**Authors:** Zahra Alahmed, Felipe Lobelo

**Affiliations:** 1 Public Health, Ministry of Health, Eastern Province, Saudi Arabia; 2 Hubert Department of Global Health, Rollins School of Public Health, Emory University, Atlanta, Georgia, United States of America; 3 Exercise is Medicine Global Research and Collaboration Center, Emory University, Atlanta, Georgia, United States of America; Aga Khan University, KENYA

## Abstract

**Background:**

Physical inactivity is a major health issue in Saudi Arabia (SA). Being physically active can improve overall health and prevent the risk of noncommunicable diseases and their consequences. The objective of this study was to assess the knowledge, attitudes, and practices of primary health care physicians in SA toward physical activity (PA) and identify the correlates of PA counseling provided by physicians.

**Methods:**

A cross-sectional study was conducted in four main cities of Eastern Province, SA (Al-Khobar, Dammam, Qatif, and Safwa). A total of 147 physicians (44%) filled out self-reported surveys that were used in the assessment of the aims.

**Results:**

Overall, 59.9% of the physicians believed that PA promotion to patients was their responsibility, and 53.7% of the physicians felt confident in their ability to provide PA counseling. However, the physicians indicated that lack of time, inadequate referral services for PA, and inadequate training in PA counseling are barriers to providing PA counseling to their patients. Only 5.4% of the physicians had excellent knowledge about PA recommendations. Female physicians were more likely to promote PA than male physicians (OR, 3.72; 95% CI, 1.10–12.58; P = .03) and more likely to assess PA in pediatric patients (OR, 2.61; 95% CI, 1.03–6.61; P = .04). Compared to other specialties, family physicians were more likely to provide general PA counseling to patients without chronic diseases (OR, 8.86; 95% CI, 1.86–42.13; P = .006). Physicians who saw fewer adult patients were more likely to systematically track/follow-up on the PA of patients (OR, 3.13; 95% CI, 1.14–8.58; P = .03) and to promote PA to pediatric patients (OR, 2.87; 95% CI, 1.37–6.00; P = .005).

**Conclusions:**

Training medical staff in PA counseling and strengthening the health care workforce and infrastructure can help physicians improve their counseling practice.

## Introduction

Physical inactivity in Saudi Arabia (SA) is considered a major public health issue. According to the WHO Diabetes Country Profile 2016 [1], 58.5% of the Saudi adult population (52.1% of males and 67.7% of females) did not meet the international physical activity (PA) recommended levels. The estimated population attributable fraction in SA, calculated with adjusted relative risks, has been reported to be 18.4% for all-cause mortality associated with physical inactivity [2].

According to JAMA, in the national physical activity plan, the American Heart Association has emphasized that “the advice from health care professionals significantly influences adoption of healthy lifestyle behaviors, including regular PA, and can increase satisfaction with medical care” [3].

There is evidence that PA counseling can be an effective strategy for reducing cardiometabolic risk factors and for the prevention and management of noncommunicable diseases (NCD), especially when offered in primary care settings [2, 4–6]. Despite the high prevalence of inactivity-related NCDs in SA (coronary heart disease, 20.4%; diabetes, 14.1%; colon cancer, 20.4%; breast cancer, 19.9%) [2], few studies have characterized PA counseling in health care settings in SA. The majority of studies available are limited to specific regions, primary health care (PHC) specialties (i.e., family physicians), or types of patients (i.e., patients with chronic disease) [7, 8]. Moreover, these studies lack specific data concerning the PA counseling correlates that may be amenable to interventions. Assessing the factors that could affect PA counseling practice is an essential step in implementing effective interventions and policies that encourage PA and will help in the identification of the barriers faced when offering PA counseling in SA’s health care setting.

The objective of this study was to explore the association between knowledge, attitudes, and practices among PHC center physicians and their provision of PA counseling. The study also evaluated the effect of the physicians’ lifestyles, personal health status, and other demographic characteristics on the provision of PA counseling.

## Materials and methods

### Study design and sample

This cross-sectional study was conducted in the four main cities of Eastern Province, SA, (AL-Khobar, Dammam, Qatif, and Safwa) between May 2016 and July 2016. Eastern Province had a population of three million and belongs to a group of provinces with the largest population among thirteen Provinces in SA [9]. A nonprobability method was used to select the sample population. This method relied on data collection from the population available to participate in the study. All physicians working in PHC centers were invited to participate in the study except dentists because their scope of service only covers the provision of dental treatment and management. The questionnaire was distributed online through social media (e.g., WhatsApp, Twitter, Facebook, LinkedIn, and email). A PA counseling guide was provided upon completion of the survey as an incentive to the participants.

The total number of PHC physicians in Eastern Province was 637, with 334 working in PHC centers in Safwa, AL-Khobar, Dammam, and Qatif [10].

### Survey

A self-reported survey developed by the National Institute of Health (NIH) (the Physician Survey of Practices on Diet, Physical Activity, and Weight Control) [11] was adapted and modified to assess the PA counseling practices provided by primary care physicians.

The survey was created using SurveyMonkey and was tested after modification using a small paper-based pilot study to ensure that the questions were understood and to assess the time required to complete the survey. This pilot survey was completed by five physicians working in PHC centers selected by the primary investigator. The survey required approximately 15–20 minutes to complete. Only one question needed modification. Since the physicians that participated in the pilot study were not from the same targeted area in Saudi Arabia, the pilot study data were excluded from the analysis.

The survey used in this study is presented in S1 Survey.

### Independent variables

Four groups of variables were assessed: i) physician demographics (age, gender, nationality, sector, marital status); ii) training (specialty, university location, knowledge about current international guidelines, and recommendations for PA); iii) physician personal health status and lifestyle behaviors including PA, sleeping hours, general health status, physician chronic disease status, and smoking status; and iv) clinical practice characteristics (PHC location, number of patients, methods used for assessing patient PA levels, frequency with which different methods of PA counselling were being provided).

### Outcome variables

Outcome variables included physician attitudes and beliefs regarding the provision of PA counseling and the frequency with which PA counseling was provided to various clinical populations (adult, children, pregnant women, and those with chronic disease).

### Ethics approval and consent to participate

The Emory Institutional Review Board (IRB) determined that this study did not require IRB review because it did not meet the definition of "research" with human subjects as outlined in Emory policies and procedures and federal rules. Approval was also obtained from the research committee of the Ministry of Health (MoH) in Eastern Province, SA, to perform the study in the MoH PHCs.

Written consent was included on the first page of the survey and obtained from all the participants. The survey did not contain any identifying information and was labeled by serial number only. The online survey was conducted securely via SurveyMonkey without collecting responder IP addresses. The data collected via paper surveys were stored in sealed envelopes.

### Data management and analysis

Data analysis was conducted using SAS software (SAS Institute Inc. 2016. SAS 9.4). Incomplete surveys were excluded from the analysis.

Descriptive statistical analysis was performed after recategorizing all variables. For continuous variables, the median was considered as the defining point of categorization.

A chi-square test was conducted to test the association between physician specialty and the frequency of PA counseling practice (providing general counseling, providing verbal counseling, providing written counseling, referring patients for further assessment and management, and following up with and tracking patients). The correlation was considered significant if p < 0.05. Fisher’s exact test was performed for the correlation of variables with small sample sizes.

Stepwise selection binary logistic regression analysis was used to examine the relationship between physician characteristics (i.e., demographics, knowledge and training, and personal lifestyle behaviors) and the frequency of PA counseling practice. For each counseling practice, one binary logistic regression model (‘always + often’ versus ‘all other levels’) was estimated, and the likelihood was computed as the predicted probabilities.

We assessed the model fitness using Hosmer-Lemeshow Goodness-of-Fit (HL- GOF) tests. A non-statistically significant result on the HL- GOF test indicate a model with a good fit. In addition, we checked the variance inflation factors (VIF) for evidence of multicollinearity and no evidence was found for all variables.

## Results

### Participants characteristics

**Fig 1:**
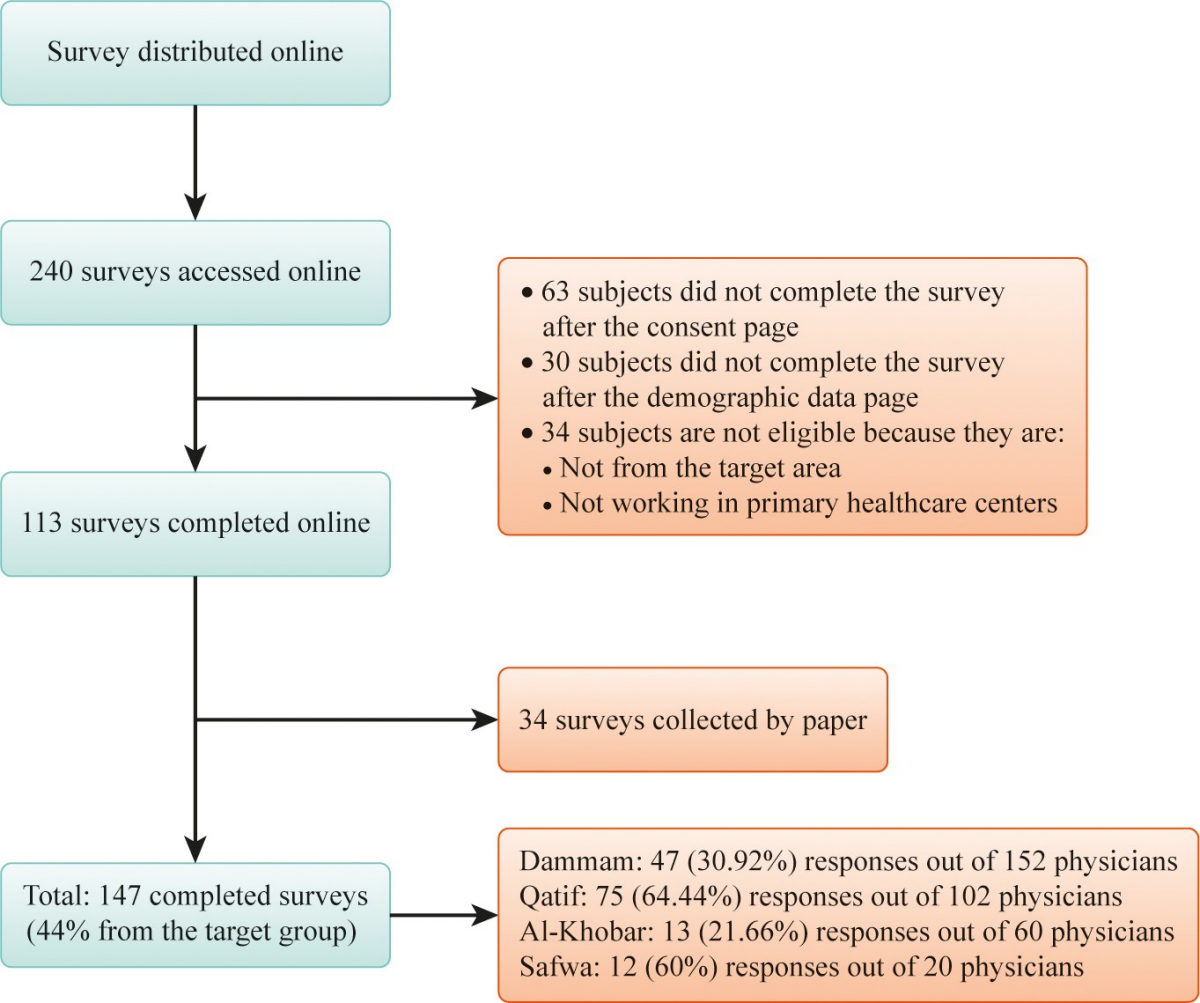
Data collection procedure.

A total of 147 PHC physicians responded to the survey, with a response rate of 44.0% (147 out of 334 physicians completed the survey). Fig 1 shows the response rate by sector and the data collection procedure; 51.0% of the respondents were from the Qatif sector, 92.5% were from Saudi, 70.8% were female, 87.1% were married, and 55.1% were below the age of 31 years (median of the sample). Nearly half of all participating physicians were general physicians, and more than 40.0% were family physicians. The Bachelor of Medicine and Bachelor of Surgery (MBBS) qualification was held by more than half of participants, and more than 71.4% had graduated from a domestic university or residency program (Tables 1 and 2).

**Table 1:** Demographic characteristics of PHC physicians.

	Number (N = 147)	Percentage (%)
**Age**		
26–31 years	81	55.1
32–60 years	60	40.8
Missing (no response)	6	4.1
**Gender**		
Male	43	29.3
Female	104	70.8
**Nationality**		
Saudi	136	92.5
Non-Saudi	11	7.5
**Marital status**		
Single	13	8.8
Married	128	87.1
Other	6	4.1
**Sector**		
Al-Khobar	13	8.8
Dammam	47	32.0
Qatif	75	51.0
Safwa	12	8.2
**Physician specialty**		
Family physician	66	45.0
General physician	69	46.9
Other specialty	12	8.2
**Education**		
Board-certified	35	23.8
Diploma	34	23.1
MBBS	76	51.7
Other	2	1.4

PHC, primary health care; MBBS, Bachelor of Medicine and Bachelor of Surgery

**Table 2:** Training and practice characteristics of PHC physicians.

	Number (N = 147)	Percentage (%)
**Years of experience after graduation from medical school**		
Less than five years	88	59.9
Five years or more	59	40.1
**Physical activity training in medical school**		
Yes	32	21.8
No	115	78.2
**University where medical degree was awarded**		
Domestic	105	71.4
International	20	13.6
Missing (no response)	22	15.0
**Physician knowledge of physical activity**		
Poor knowledge	87	59.2
Good knowledge	49	33.3
Excellent knowledge	8	5.4
Missing (no response)	3	2.0
**Average number of adult patients seen by the physicians per day**		
20 or fewer	85	57.8
More than 20	59	40.1
Missing (no response)	3	2.0
**Average number of pediatric patients seen by the physicians per day**		
10 or fewer	81	55.1
More than 10	61	41.5
Missing (no response)	5	3.4
**Number of physicians in PHC**		
4 or fewer	35	23.8
More than 4	112	76.2
**Number of nurses in PHC**		
Less than 15 nurses	110	74.8
15 nurses or more	37	25.2
**Electronic referral system in PHC**		
Yes	44	29.9
No	103	70.1

**Domestic:** King Abdul-Aziz University, King Faisal University, King Saud University, Qassem University, University of Dammam; **International:** Arabian Gulf University, Beirut University, Cairo University, Jordan University of Science and Technology, Karachi University, Shendi University, Vienna University; **Poor level of knowledge:** knowing 0–1 recommendation; **Good level of knowledge:** knowing 2–3 recommendations; **Excellent level of knowledge:** knowing 4–6 recommendations), PHC, primary health care.

Only 21.8% had received training about PA counseling during medical school or residency program. A small proportion (5.4%) had excellent level of knowledge about PA guidelines and recommendations. Regarding the physicians’ health status, 24.0% reported having one or more chronic diseases, while 43.0% reported having a very good general health status. Overall, 57.8% of participants saw 20 or fewer adult patients per day, and 55.1% observed 10 or fewer pediatric patients per day. Of physicians and nurses working in PHC centers, 76.2% had 4 or more physicians working at the same PHC center, and 25.2% had at least 15 nurses working at the same PHC center. Approximately 30% reported having an electronic referral system (ESR).

In total, 43.0% of the physicians were found to be either overweight or obese, and only 17.3% complied with the recommended hours of sleep per night, according to the American Academy of Sleep Medicine (AASM) guidelines. Only 7.9% of the physicians had the habit of smoking. Overall, 77.5% reported the practice of PA. However, only 27.1% practiced the recommended aerobic activity for adults according to the guidelines [moderate PA (150 min/week) and vigorous PA (75 min/week)]. About a quarter (27.5%) of the physicians reported that they do strength- building PA.

The majority (59.9%) of the physicians indicated the physician as the main health professional responsible for promoting PA, followed by the physical education professionals.

Furthermore, 58.5% agreed that they were effective in helping patients to be adequately physically active, and 53.7% indicated that they were confident about their ability to do so. In addition, 84.4% of the physicians agreed that they were able to provide more credible and effective counseling if they were adequately physically active themselves.

**Fig 2:**
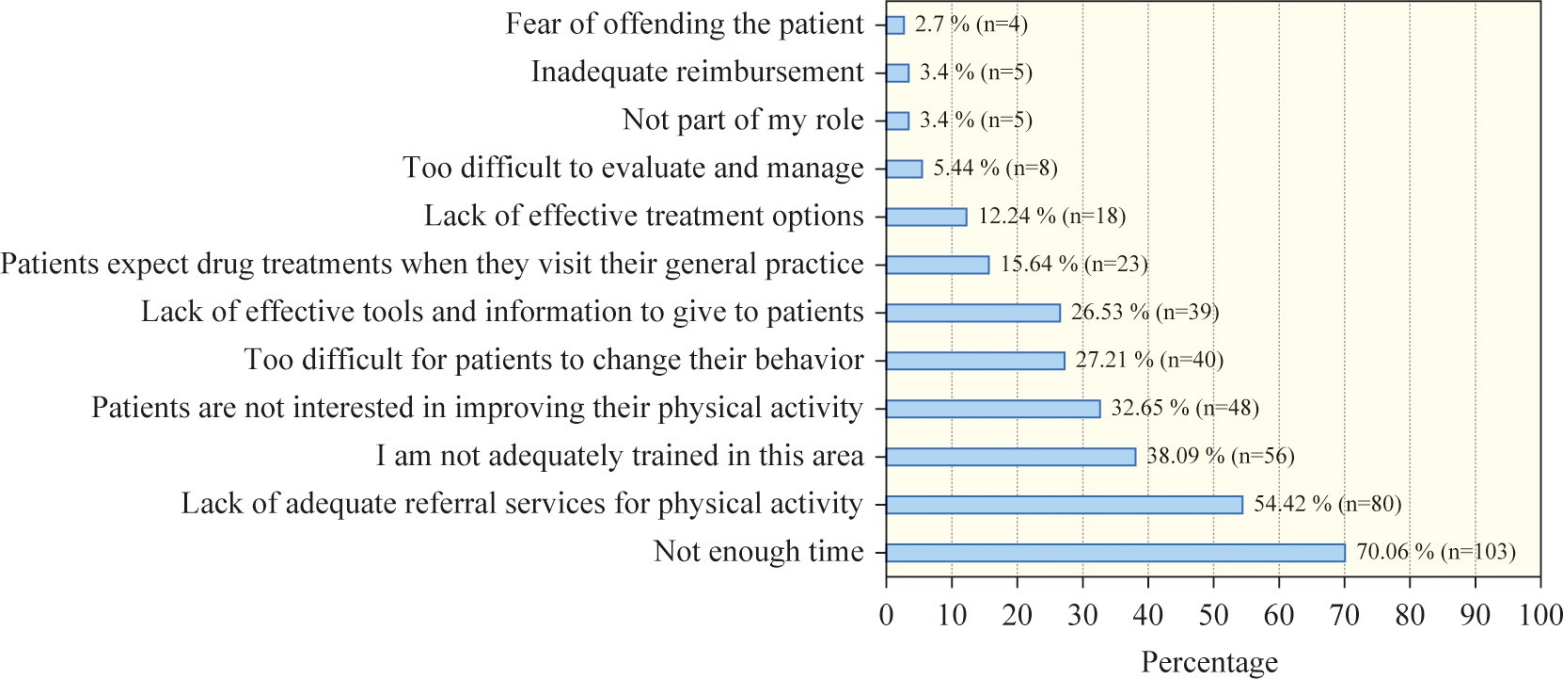
Percentage of perceived barriers to providing physical activity counseling from the physicians’ perspective.

The top three barriers reported by the physicians that prevented them from providing PA counseling to their patients were the following: lack of time, lack of adequate referral services for PA, and lack of adequate training in PA (70.1%, 54.4%, and 38.1%, respectively; Fig 2).

### Association analysis

Stepwise selection binary logistic regression analyses were used to examine the association between various physician characteristics and PA counseling practice. For each counseling practice, one binary logistic regression model (‘always + often’ versus ‘all other levels’) was estimated, and the likelihood was computed as the predicted probabilities. Model fit was assessed for all models using Hosmer-Lemeshow Goodness-of-Fit tests. A non-statistically significant result on the H-L GOF test represents a good-fitting model (P value range from 0.1–0.9) In addition, we checked the VIF for evidence of multicollinearity and no evidence was found for all variables, the all VIF values were less than 2.

#### Association between physician specialty and the frequency of physical activity counseling practice

Different types of PA assessments were used (general questions about the amount of PA, Specific questions about the duration, intensity, and type of PA, Standardized PA questionnaire, pedometer, and other technologies such as phone application, tablets, fitbit). There was a statistically significant difference in the use of specific questions about the duration, intensity, and type of PA as well as the use of other technology in the assessment of PA between family and general physicians (p < 0.05).

With regard to the frequencies of assessing PA in and promoting PA to the adults, children, and pregnant women, family physicians were more likely to assess and promote PA to adult patients (p < 0.05) compared to the general physicians

Among the specialists working in PHC centers, family physicians reported the highest prevalence of providing general counseling to patients with (93.9%) and without (45.5%) chronic disease, and 95.5% provided verbal behavioral counseling to patients with chronic disease. Among all the family and general physicians, the prevalence of providing PA counseling with all the associated modalities increased if the patient had chronic disease.

A chi-square test was conducted to demonstrate the statistical difference between physician specialties and their PA counseling practice. There was a statistically significant difference between specialties in the referral of patients without chronic diseases for further assessment and the management of PA. Family physicians differed significantly from other specialists in the frequency of providing general counseling and verbal behavioral counseling as well as in following up and tracking patients with chronic diseases.

#### Characteristics associated with physicians’ PA counseling practice

Female physicians were more likely than male physicians to provide general PA counseling to patients without chronic disease (odds ratio [OR], 1.88; 95% confidence interval [CI], 0.75–4.74) and to assess PA in adult patients (OR, 1.66; 95% CI, 0.80–3.45), but the differences were not statistically significant. Furthermore, female physicians were also more likely to promote PA than male physicians, and this difference was statistically significant (OR, 3.72; 95% CI, 1.10–12.58; p = 0.03). Finally, female physicians were more likely to assess PA in pediatric patients than male physicians, and this difference was also statistically significant (OR, 2.61; 95% CI, 1.03–6.61; p = o.04).

Physicians who were aged ≥ 31 years were less likely to assess and promote PA in pregnant women (OR: 0.41, 95% CI 0.15–1.13, p = 0.08), (OR: 0.18, 95% CI 0.04–0.86, p = 0.03), respectively. In addition, they were less likely to refer patients without chronic disease for further evaluation and management for PA (OR: 0.44, 95% CI 0.21–0.93, p = 0.03).

Compared to other specialties, family and general physicians were more likely to provide general PA counseling to patients without chronic diseases (OR, 8.86; 95% CI, 1.86–42.13; p = 0.006; and OR, 7.13; 95% CI, 1.60–31.89; p = 0.01, respectively). Moreover, family and general physicians were more likely than physicians in other specialties to promote PA to pregnant women, but this difference was not statistically significant.

Physicians who had graduated from domestic universities were less likely to provide general counselling, written prescription, or systematically track/follow-up PA in patients without chronic disease (OR, 0.39; 95% CI, 0.14–1.13; p = 0.08), (OR, 0.34; 95% CI, 0.09–0.1.33; p = 0.12), (OR, 0.33; 95% CI, 0.11–0.99; p = 0.05), respectively, than were physicians who had graduated from international universities. In contrast, this difference was not statistically significant for patients with chronic disease. In addition, graduates from domestic universities were statistically less likely to promote PA to adult patients (OR, 0.12; 95% CI, 0.017–0.80; p = 0.03) and less likely to promote PA to pediatric patients (OR, 0.62; 95% CI, 0.23–167; p *=* 0.13) than were those who had attended international universities. Moreover, physicians who graduated from domestic universities were more likely to assess (p = 0.01) and promote PA (p = 0.002) among pregnant women than physicians who graduated from international universities. Physicians who received training in medical school or followed a PA counseling specialty program were more likely to assess PA in adults than those who had not received such training (p = 0.04). Physicians who were board certified or held a diploma degree were less likely to refer patients without chronic diseases for further evaluation and management of PA than were physicians with an MBBS.

Physicians with good or excellent level of knowledge of PA guidelines and recommendations were more likely to promote PA in pediatric patients, and those with excellent level of knowledge were more likely to assess PA in pregnant women (OR, 5.16; 95% CI, 0.68–39.36).

The number of adult patients seen by physicians per day was found to affect PA counseling practice; physicians who saw fewer adult patients were less likely to provide general counseling to patients without chronic disease (OR, 0.33; 95% CI, 0.15–0.72; p *=* 0.005) but were more likely to systematically track/follow-up on the PA of patients (OR, 3.13; 95% CI, 1.14–8.58; p = 0.03) and to promote PA to pediatric patients (OR, 2.87; 95% CI, 1.37–6.00; p = 0.005).

Physicians with good to excellent general health status were more likely to provide general PA counseling to patients without chronic disease but were less likely to systematically track/follow-up on the PA of chronic disease patients or to promote PA to adult patients. These findings were not statistically significant.

Similarly, physicians without chronic disease were more likely to provide written prescriptions for PA to patients with chronic diseases and to assess and promote PA in pediatric patients. However, none of these findings were statistically significant.

Finally, if physicians met the recommendation of seven or more hours of sleep per night, they were more likely to assess PA in pregnant women (OR, 4.42; 95% CI, 1.12–17.35; p *=* 0.03).

Physicians from Safwa and Al Khobar sector were more likely to promote PA to adult patients. On the other hand, physicians from Dammam sector were less likely to do so. In addition, physicians from all sectors were less likely to assess PA in pregnant women. Further results are summarized in Tables 3 and 4.

**Table 3:** Physician lifestyle characteristics associated with physical activity counseling practice.

Physician lifestyle characteristic	Physical activity counseling practice	OR (CI)	P
**Physicians who had no chronic disease**	Provide general counseling to patients without chronic disease	0.46 (0.19–1.11)	0.08
	Provide written prescriptions to patients with chronic disease	2.22 (0.75–6.53)	0.18
	Assess physical activity in pediatric patients	2.54 (0.10–6.79)	0.06
	Promote physical activity to pediatric patients	1.32 (0.55–3.13)	0.53
**Non- smoker physicians**	Provide verbal behavioral counseling to patients without chronic disease	0.31 (0.06–1.52)	0.14
**Physicians with good general health status**	Provide General counseling to patients with chronic disease	4.08 (0.68–24.32)	0.12
	Systematically track/follow up patients with chronic disease	0.28 (0.08–0.95)	0.04
	Promote physical activity in adult patients	0.42 (0.10–1.76)	0.23
**Physicians with excellent general health status**	Provide general counseling to patients with chronic disease	1.66 (0.44–6.30)	0.46
	Systematically track/follow up patients with chronic disease	0.33(0.10–1.07)	0.06
	Promote physical activity to adult patients	0.66 (0.15–2.86)	0.57
**Physician whose sleeping hours were ≥ 7 hours**	Assess physical activity in pregnant women	4.42 (1.12–17.35)	0.03
**Physicians who practiced moderate-to- vigorous activity (≥ 150 minutes/week|)**	Promote physical activity to adult patients	1.80 (0.45–7.13)	0.4

Stepwise selection binary logistic regression analysis was used to examine the association between various physician characteristics and PA counseling practice. For each counseling practice, one binary logistic regression model (‘always + often’ versus ‘all other levels’) was estimated, and the likelihood was computed as the predicted probabilities.

OR, odds ratio; CI, confidence interval.

**Table 4:** Association between physicians setting characteristics and physical activity counseling practice.

Physicians setting characteristics	Physical activity counseling practice	OR (CI)	P
Physician who see ≤ 20 patients or per day, among adult patients	Provide general counseling to patients without chronic disease	0.33 (0.15–0.72)	0.005
	Provide written prescriptions to patients without chronic disease	0.27(0.07–1.07)	0.06
	Systematically track/follow up patients without chronic disease	3.13 (1.140–8.58)	0.03
	Provide verbal behavioral counseling to patients with chronic disease	0.24 (0.05–1.16)	0.08
	Promote physical activity to pediatric patients	2.87 (1.37–6.00)	0.005
Physicians who see ≤ 10 patients per day, among pediatric patients	Refer patients without chronic disease for further evaluation and management	3.98 (1.36–11.70)	0.01
	Promote physical activity to adult patients	0.26 (0.09–0.78)	0.02
Physicians who worked with ≥ 15 nurses per PHC	Provide verbal behavioral counseling to patients with chronic disease	0.27 (0.08–0.91	0.04
	Provide written prescriptions to patients with chronic disease	0.29 (0.08–1.01)	0.05
	Systematically track/follow up patients with chronic disease	0.1 (0.03–0.31)	<0.001
	Promote physical activity to adult patients	0.33 (0.11–1.03)	0.06
	Assess physical activity in pregnant women	0.29 (0.08–1.07)	0.06
Physicians who worked with > 4 physicians per PHC	Promote physical activity to adult patients	0.41 (0.13–1.27)	0.12
	Assess physical activity in pediatric patients	1.56 (0.66–3.70)	0.31
Physicians who had > 5 years of experience	Promote physical activity to adult patients	0.44 (0.15–1.32)	0.14
	Promote physical activity to pregnant women	3.81(0.82–17.60)	0.09
Physicians who had electronic referral system in the PHC	Assess physical activity in pregnant women	6.34 (1.79–22.47)	0.11

Stepwise selection and binary logistic regression were used to examine the association. For each counseling practice, one binary logistic regression model (‘always + often’ versus ‘all other levels’) was estimated, and the likelihood was computed as the predicted probabilities.

OR, odds ratio; CI, confidence interval; PHC, primary health care

#### Characteristics associated with physician attitudes toward PA counseling

The presence of an ESR and physicians’ chronic disease status affected the response to the statement that “It is important that PA programs for the community be offered by the health system.”

Physicians who slept for the recommended number of hours per night, who had no chronic disease, had good to excellent level of knowledge of PA recommendations, or worked in the Safwa, Al-Khobar, or Dammam sectors were more likely to agree that there are effective strategies and/or tools to help patients achieve adequate PA.

Physicians who were female, had good to excellent level of knowledge of PA recommendations, were ≥ 31 years old, had a specialty in family medicine, observed fewer adult patients (< 20 patients/day), or had ≥ 15 nurses working in their PHC center, were more likely to report that they were confident in their ability to counsel patients to be physically active.

Moreover, physicians who were female, had good to excellent level of knowledge of PA recommendations, had ≥15 nurses working in their PHC center, or were family or general physicians felt that they were effective at helping patients to achieve adequate PA.

Finally, physicians believed that a physician/nurse would be able to provide more credible and effective counseling if he/she achieved adequate PA, was female, had ≥ 15 nurses working in their PHC center, was a family or general physician, or was ≥ 31 years old.

## Discussion

The present research is the first study in Eastern Province, SA, to assess PA counseling practice among all physician specialists (except dentists) working in PHC centers and that also assessed whether the knowledge or characteristics of physicians had effects on PA counseling. Therefore, the findings of this study may be used to improve the quality of care provided in PHC centers and to improve health promotion services (especially those related to lifestyle modification) as a mode of health promotion and disease prevention in SA.

Almost 60% of the physicians we surveyed indicated the physician as the main health professional responsible for promoting PA counseling. This result corresponds to the findings by Buffart (2009), who reported that 98% of the general physicians agreed that promoting PA among patients is part of their role [12, 13]. This result also corroborates the finding of Van der Ploeg (2007) that 92–99% of general physicians agreed that suggesting methods to increase daily PA to patients is part of their role[12, 14]. Graham (2005) also found that the majority of general physicians felt that the promotion of PA was part of their role [12, 15]. However, although it should be one of the main roles of physicians to promote PA, other health care workers, such as nurses, should also be properly trained in PA counseling. Training of nurses and other health care workers to provide PA counseling can potentially decrease the responsibility of physicians, who already have high patient numbers without added counseling duties. Therefore, if more health care workers were to be trained, patients would be more likely to receive PA counseling.

In addition, this study found that physicians both believed in the importance of PA programs for the community and felt competent and confident about their ability to help patients in being adequately physically active. This result was similarly reported in a systematic review of provider perceptions of PA counseling, with three studies demonstrating that 66–92% of physicians felt confident in providing general counseling about PA to patients [12]. However, although the physicians surveyed in this study were confident of their ability to provide physical counseling, 59.2% of the physicians were found to have poor level of knowledge regarding recommended PA guidelines. This can be explained by the fact that 38.1% reported inadequate training in PA counseling and that only 21.8% received training on PA counseling during medical school or a specialty program. The results of these previous studies, as well as our own results, is consistent with those of a cross-sectional study conducted in Jeddah, SA, showing that primary care physicians were not aware of current PA guidelines [16]. Such results suggest the need to modify the training curriculum for medical schools and specialty programs by adding sections regarding the promotion of services for lifestyle changes. In other words, education needs to focus on how to provide PA counseling.

Lack of time, lack of adequate referral services for PA, and inadequate training were the top three barriers that physicians identified as preventing them from providing PA counseling to patients. Some of these barriers were found in other studies, with 14 indicating that lack of time is the biggest barrier to PA counseling and 8 indicating that lack of knowledge and training in PA counseling are the greatest barriers [12]. Furthermore, these two barriers were also f the most common barriers reported by primary care physicians in a study conducted in the Asser Region, SA [7].

To overcome these barriers, PA counseling training programs should be implemented by the MoH for medical staff working in PHC centers to help improve physical counseling practice. Additionally, lifestyle counseling and health coaching need to be added to the medical school curriculum. To address this issue, SA should enhance the health care infrastructure by increasing the number of available physicians and medical staff with PA training in each PHC center such that physicians will be able to spend more time on lifestyle counseling. Furthermore, efforts should be made to work with communities to develop ERSs for community centers and gyms as well as secondary health care centers (e.g., hospitals and specialist centers) to promote PA. ERSs are the pathway to the more specialized assessment and management of patient health issues. For example, secondary health care workers (e.g., PA therapists) would be able to gain specific and specialized insight into patient issues and conduct further PA assessment and management. With regard to community centers and ERSs, ERSs would provide a potentially cost-effective resource to promote PA in patients since the physicians would be able to help in ensuring that patients perform PA under the supervision of physical trainers who could also ensure the prescribing and monitoring of PA according to patient needs [17, 18]. A combination of these approaches regarding physical counseling in PHC centers could potentially help decrease physical inactivity in SA and its related health and economic burdens.

In our sample, family physicians were the most likely health care workers to provide PA counseling, especially if patients had chronic diseases. This finding was supported by Al-Shammari (2016), who explored whether family medicine residents in Eastern Province counsel chronic disease patients about PA [8]. In addition, a significant difference was seen in our sample between family and general physicians regarding their assessment of PA through the use of specific questions about the duration, intensity, and type of PA. These findings can be attributed to family physicians’ knowledge as well as the training they received during specialty residency programs on the importance of lifestyle changes in the management of chronic diseases and disease prevention. Therefore, given the importance of residency programs in providing PA counseling, each SA physician working in PHC should also undergo PA counseling training during their residency program.

Several factors and characteristics were associated with physicians’ attitudes toward PA counseling and their counseling practice, including physician age, gender, specialty, education level, university of graduation, level of knowledge about PA guidelines, general health status, sleeping habits, PHC sector, number of patients seen per day, number of nurses working in the PHC center, and the availability of an ERS. Gender and specialty have previously been identified in studies conducted in the United States as factors associated with the PA practices of primary care physicians [19].

According to this study’s results, the physicians also believes in their ability to provide more credible and effective counseling if they were adequately physically active. However, based on the survey results, more than 40% of the physicians were overweight or obese, with only 19.1% meeting the global PA (aerobic + strength) recommendations. In fact, four studies done in SA in other regions than this study found that health care professionals and physicians in residency programs were either physically inactive or had low level of PA [7, 20–22]. This lack of PA could be due to the very busy working hours related to the shortage of physicians, which might have led to the high daily patient load for each physician. Also, that 70% of our sample were female could have contributed to this lack of PA among physicians. Being female in Saudi culture implies having other responsibilities at home (e.g., raising children and housework) that could prevent them from achieving the recommended minutes of PA per week for most of them. Therefore, to increase physicians’ PA levels, the MoH should mandate a one-hour break during working hours to practice some sort of PA by each physician. However, this study did not find statistical difference in physician PA counseling practice in relation to their own PA levels, which is in contrast to the findings of two studies in southern (sample size = 106) and southwestern (sample size = 232) regions of SA, showing a significant relationship between physician PA and their PA counseling practice [7, 23]. The difference in this present results compared to the 2 previous studies may be as a result of the study sample size, with a response rate of 44.0% (147 from 334 physicians) compared to the previous studies response rate ranging from 64–77%. This conflicting results needs further examination in future studies. Moreover, two scientific papers indicated that primary care physicians who practiced healthy lifestyle behaviors will be role models and will have the ability to provide better and effective lifestyle counseling to their patients, thereby motivating them [24, 25].

**Fig 3:**
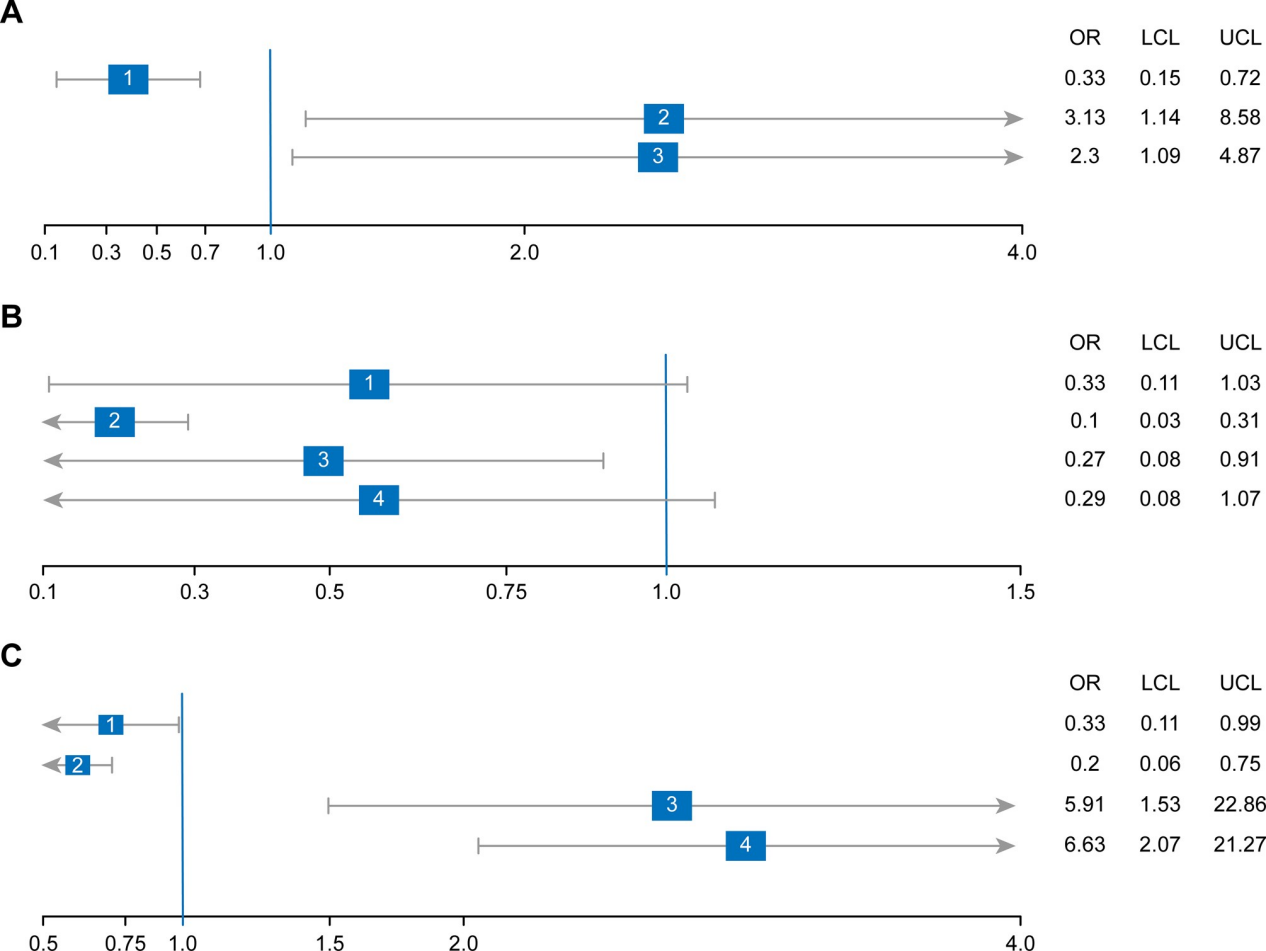
Characteristics affecting PA counseling practice. PA: physical activity, OR: odds ratio, LCL: lower confidence limit, UCL: upper confidence limit. Part A. Outcomes affected by the number of adult patients (or fewer patients vs. more patients) 1: Physicians provide verbal behavioral counseling for patients without chronic disease. 2: Physicians systematically track/follow up patients without chronic disease. 3: Physicians promote physical activity in pediatric patients. Part B. Outcomes affected by the number of nurses in PHC center (or fewer nurses vs. more nurses) 1: Physicians promote physical activity in adult patients. 2: Physicians systematically track/follow up patients with chronic disease. 3: Physicians provide verbal behavioral counseling for patients with chronic disease. 4: Physicians assess physical activity for pregnant women. Part C. Outcomes affected by the university of graduation (or domestic vs. international university) 1: Physicians systematically track/follow up patients without chronic disease. 2: Physicians systematically track/follow up patients with chronic disease. 3: Physicians assess physical activity in pregnant women. 4: Physicians promote physical activity in pregnant women.

We expected that physicians who observed fewer adult patients per day and had more nursing staff would more frequently provide counseling to patients, regardless of chronic disease status. However, we found that physicians who observed fewer adult patients per day were less likely to provide verbal counseling to patients without chronic disease. This finding was not consistent with the information reported by physicians in this study. Participants reported that the high number of patients resulted in insufficient time to provide PA counseling. Further studies are required to address the discrepancies in these results. However, it should be noted that physicians who attend to fewer adult patients per day were more likely to systematically track and follow up on PA in chronic disease patients and to promote PA to children (see Fig 3: part A).

Physicians with more nursing staff were less likely to provide verbal counseling and systematically track/follow up patients with chronic disease (see Fig 3: part B). Physicians may rely on nursing staff to provide this service when a greater number of nurses work in the center. Therefore, training of nursing staff may be required to ensure adequate provision of PA counseling. In addition, in the current study, physicians indicated feeling more confident and competent when counseling patients about PA if there were more nursing staff in their PHC center.

Physicians who graduated from international universities were found to assess and promote PA for pregnant women less than physicians who graduated from domestic universities. Physicians who graduated from international universities might have assumed that due to Saudi culture and social norms, it is unnecessary to assess and promote PA to pregnant women because they do not go outside the home to engage in PA alone. However, physicians who graduated from international universities systematically track/follow up on the PA of patients more frequently, regardless of chronic disease status (see Fig 3: part C). This suggests that domestic and international medical school curriculums may vary in content. Therefore, domestic universities in SA need to develop a more effective curriculum, and collaborate with international universities.

### Limitations

One limitation of this study is the sampling method; thus, it is not possible to generalize the results on a national scale. However, the selected sample pool provided valuable insight into PA counseling practices in the Eastern Province, as it represented the largest physician specialist group in that area.

The study involved a self-reported survey; therefore, the reported PA counseling practices may not reflect the actual practice of physicians; and physicians may overestimate the effectiveness of their practices. Future prospective research measuring the impact of PA counseling is required.

The survey time length may be considered a limitation and may have led to a decrease in the response rate and number of complete surveys collected. However, this time length was necessary to provide an in-depth evaluation of the knowledge, attitudes, and PA counseling practices of physicians.

## Conclusion

The results of this study suggest the need to improve physician and health care workers’ knowledge and counseling skills through PA counseling training program. Furthermore, national practical PA guidelines should be distributed to all PHC centers. In addition, to allow for adequate PA counseling time by physicians, it is necessary to strengthen the PHC center workforce and infrastructure. Moreover, to promote PA and coordinate a referral system, focused collaboration between PHC and community centers is needed. Additionally, mandatory PA assessment questions could be included when performing vital sign checks during PHC visits. Furthermore, PA assessment forms and educational materials could be designed and made available for physicians’ use.

Altogether, these approaches may promote PA among the Saudi population and decrease economic and health-related burdens of physical inactivity.

Long-term studies on the actual effect of PA counseling among the Saudi population are necessary to develop effective strategies and approaches to promote the adoption of PA counseling within PHC centers. Such studies could help measure whether patients’ health outcomes and PA behaviors are improved with counseling. Promotion and approaches to increasing PA counseling may decrease the economic and health-related burden of physical inactivity in the Saudi population.

## Supporting information

S1 Survey (DOCX)
