# Supplementary material for: Correlates of physical activity counseling provided by physicians: A cross-sectional study in Eastern Province, Saudi Arabia
Source: PLoS One. 2019 Jul 25;14(7):e0220396. doi: 10.1371/journal.pone.0220396 (PMC6657910; doi:10.1371/journal.pone.0220396)
Supplement: S1 Survey — (DOCX) [file pone.0220396.s001.docx]

Survey

**Dear respondent,**

This questionnaire Is conducting to study the Knowledge, Attitude and Practices of health care practitioner in Saudi Arabia Towards physical activity counseling and its correlates .

**Participation in this study is voluntary, if you decide to participate we will ask you to sign consent.** **the following questions and the information gathered through this questionnaire will be confidential and used for health services improvements**. **However, you are free to withdraw from the study at any time, without providing any justification for that. This questionnaire will take about 15-20 minutes of your time.**

- **I agree to participate in this study**

**Signature:**

**Questionnaire number #:**

**Section A: Demographic data**

| **Age in years** |  | | | | | | |
| --- | --- | --- | --- | --- | --- | --- | --- |
| **Gender** | Male | | Female |  | | | |
| **Nationality** | Saudi | | Non-Saudi |  |  |  |  |
| **PHC Sector** |  | | | | | | |
| **Marital Status** | single | | Married | Divorced | other | | |
| **Doctor Specialty** | General Physician | | Family medicine | Internal medicine | Pediatric | Oby/gyn | Other :……… |
| **Education** | Board certified | | Diploma | Master | Doctorate | Bachelor | Other :……… |
| **Years of experience after graduation from medical school** | . . . . . . . . . . . Year(s) | | | | | | |
| **Did you get training in medical school /specialty program about physical activity counseling** | Yes | How many hours : ………. | | | | | |
|  | No |  | | | | | |
| **Height** | …….cm | | | | | | |
| **Weight** | ……kg | | | | | | |

**Section B: Clinical practice**

| **Average # of Adult patients** | …………………./day |  |  |
| --- | --- | --- | --- |
| **Average # of Child/Adolescent patients** | …………………./day |  |  |
| **Number of physician in your center** | Circle the number apply to your setting | 0-1-2-3-4-5-6-7-8-9-10 |  |
| **Number of nurses in your center** | Circle the range apply to your setting | 4-6  8-10  12-14  16-18  20-22  >22 |  |
| **Is there an electronic referral system in your center** | Yes | No |  |

B1. During routine well-patient physical exams of your adult (18 years and older) patients:

***Check one in each row***

|  | **Never** | **Rarely** | **Sometimes** | **Often** | **Always** |
| --- | --- | --- | --- | --- | --- |
| How often do you assess physical activity? |  |  |  |  |  |
| As a general policy, for your entire adult patient population, how often do you promote:  Physical Activity |  |  |  |  |  |

B2. During routine well-patient physical exams of your pediatric and adolescent (2-17 years) patients:

***Check one in each row***

|  | **Never** | **Rarely** | **Sometimes** | **Often** | **Always** |
| --- | --- | --- | --- | --- | --- |
| How often do you assess physical activity? |  |  |  |  |  |
| As a general policy, for your entire pediatric patient population, how often do you promote:  Physical Activity |  |  |  |  |  |

B3. During routine well-patient physical exams of pregnant women:

***Check one in each row***

|  | **Never** | **Rarely** | **Sometimes** | **Often** | **Always** |
| --- | --- | --- | --- | --- | --- |
| How often do you assess physical activity? |  |  |  |  |  |
| As a general policy, for your entire pregnant patient population, how often do you promote:  Physical Activity |  |  |  |  |  |

B4. For your adult patients WITHOUT chronic disease (Diabetes, Hypertension, Dyslipidemia, Arthritis, chronic back pain, Cancer, coronary heart disease, Asthma) who are insufficiently active: How often do you…

***Check one in each row***

|  | **Never** | **Rarely** | **Sometimes** | **Often** | **Always** |
| --- | --- | --- | --- | --- | --- |
| Provide general counseling for physical activity? |  |  |  |  |  |
| Provide verbal behavioral counseling on Physical Activity (e.g., “Increase your exercise by walking daily”)? |  |  |  |  |  |
| Provide written prescription on Physical Activity |  |  |  |  |  |
| Refer these patients to another health professional or program outside of your practice for further evaluation and/or management?  If you refer your patient Where do you refer your patient  for further physical activity counseling/management?   - Physiotherapist - Community centers (gym) - Other:……. |  |  |  |  |  |
| Systematically track/follow patients over time concerning behaviors or other measures of progress related to physical activity? |  |  |  |  |  |

B 5. For your adult patients WITH chronic disease (Diabetes, Hypertension, Dyslipidemia, Arthritis, chronic back pain, Cancer, coronary heart disease, Asthma) who are insufficiently active, or are overweight: How often do you…

***Check one in each row***

|  | **Never** | **Rarely** | **Sometimes** | **Often** | **Always** |
| --- | --- | --- | --- | --- | --- |
| Provide general counseling for physical activity? |  |  |  |  |  |
| Provide verbal behavioral counseling on Physical Activity (e.g., “Increase your exercise by walking daily”)? |  |  |  |  |  |
| Provide written prescription on Physical Activity |  |  |  |  |  |
| Refer these patients to another health professional or program outside of your practice for further evaluation and/or management?  If you refer your patient Where do you refer your patient  for further physical activity counseling/management?   - Physiotherapist - Community centers (gym) - Other:……. |  |  |  |  |  |
| Systematically track/follow patients over time concerning behaviors or other measures of progress related to physical activity? |  |  |  |  |  |

B 6. If you assess physical activity, HOW do you assess it?

***Check one in each row***

|  | **Yes** | **No** |
| --- | --- | --- |
| General questions about amount of physical activity |  |  |
| Specific questions about duration, intensity, and type of physical activity |  |  |
| Standardized physical activity questionnaire |  |  |
| Pedometer |  |  |
| Other technology (phone application ,tablets ,fit pit …….) |  |  |

**B7. Which of the following are the TOP 3 BARRIERS to evaluating and/or managing your patients’ physical activity, in your practice?**

Check the top 3 barriers

- No enough time
- Not part of my role
- I am not adequately trained in this area
- Too difficult to evaluate and manage
- Inadequate reimbursement
- Lack of adequate referral services for physical activity
- Patients are not interested in improving their physical activity
- Fear of offending the patient
- Too difficult for patients to change their behavior
- Lack of effective tools and information to give to patients
- Lack of effective treatment options
- Patients expect drug treatments when they visit their GP practice

**Section C: Knowledge**

**C 1. According to current guidelines, for adults, 18 to 65 years, how much moderate to vigorous physical activity is recommended (minute/week) for general health and prevention of chronic diseases?**

*Check one box*

- 20 minutes
- 30 minutes
- 40 minutes
- 60 minutes
- 90 minutes
- 120 minutes
- 150 minutes
- 250 minutes
- 300 minute
- Don’t Know

**C 2 According to current guidelines, for adults, 65 years and older, how much moderate to vigorous physical activity is recommended (minute/week) for general health and prevention of chronic diseases?**

*Check one box*

- 20 minutes
- 30 minutes
- 40 minutes
- 60 minutes
- 90 minutes
- 120 minutes
- 150 minutes
- 250 minutes
- 300 minute
- Don’t Know

**C3. According to current guidelines, for children/adolescents, (2-17 years), how much moderate physical activity is recommended (minutes/every day) for general health and prevention of chronic diseases?**

*Check one box*

- 20 minutes
- 30 minutes
- 40 minutes
- 60 minutes
- 90 minutes
- 120 minutes
- 150 minutes
- 250 minutes
- 300 minute
- Don’t Know

**C4. According to current guidelines, the total amount of minutes each week of physical activity of a moderate or vigorous effort can be spread for at least how many minute at time?**

*Check one box*

- 10 minutes
- 15 minutes
- 20 minutes
- 25 minutes
- 30 minutes

**C6. According to current guidelines, for pregnant how much moderate physical activity is recommended (minutes/week) for general health and prevention of chronic diseases?**

*Check one box*

- 0 minutes/don’t recommended
- 20 minutes
- 30 minutes
- 40 minutes
- 60 minutes
- 90 minutes
- 120 minutes
- 150 minutes
- 250 minutes
- 300 minute
- Don’t Know

**C7. According to current guidelines, for obese individual how much moderate physical activity is recommended (minutes/week) for general health and prevention of chronic diseases?**

*Check one box*

- 20 minutes
- 30 minutes
- 40 minutes
- 60 minutes
- 90 minutes
- 120 minutes
- 150 minutes
- 250 minutes
- 300 minute
- Don’t Know

**Section D: attitude**

|  | **Strongly agree** | **agree** | **Natural** | **disagree** | **Strongly disagree** |
| --- | --- | --- | --- | --- | --- |
| It is important that physical activity programs for the community are offered by the health system. |  |  |  |  |  |
| There are effective strategies and/or tools to help patients: be adequately physically active |  |  |  |  |  |
| I am confident in my ability to counsel my patients to: be adequately physically active |  |  |  |  |  |
| I am effective at helping my patients: be adequately physically active |  |  |  |  |  |
| specifically, a physician /nurse will be able to provide more credible and effective counseling if he/she: is adequately physically active. |  |  |  |  |  |

**D1. Please indicate how strongly you agree with each of the following statement**

**D2. Who is the main health professional responsible for promoting physical activity?**

- Physician
- Nurse
- Physical education professional
- Nutritionist
- Physiotherapist
- Other

**Section E: Personal behaviors**

**This section about your personal behaviors related to physical activity in your daily life**

**E1. Moderate physical activities make you breathe somewhat harder than normal. During the last 7 days, did you do any moderate physical activities for at least 10 minutes? Think about activities such as bicycling, swimming, brisk walking, dancing, or gardening.**

- No Go to E2
- Yes

**a. On how many of the past 7 days did you do moderate physical activities?**

- Days

**b. In the past 7 days, on a typical day in which you did moderate physical activities, how much time did you spend doing them?**

- Minutes per day

**E2. Vigorous activities make you breathe much harder than normal. Now think about vigorous activities you did that take hard physical effort, such as aerobics, running, soccer, fast bicycling, or fast swimming. During the last 7 days, did you do any vigorous physical activities in your free time for at least 10 minutes?**

- No Go to E3
- Yes

**a. On how many of the past 7 days did you do vigorous physical activities?**

- Days

**b. In the past 7 days, on a typical day in which you did vigorous physical activities, how much time did you spend doing them?**

- Minutes per day

**E3. Now think about activities specifically designed to STRENGTHEN your muscles, such as lifting weights or other strength-building exercises. Include all such activities even if you have included them before. During the last 7 days, did you do activities to strengthen your muscles?**

- No
- Yes

**E4. Would you say that in general your health is:**

- 1 Excellent
- 2 Very good
- 3 Good
- 4 Fair

**E5. Do you have any chronic disease:**

*Check all that apply*

- Diabetes
- Hypertension
- Dyslipidemia
- Arthritis
- Chronic back pain
- Cancer
- coronary heart disease
- Asthma
- Thyroid disorders

**E6. On average, how many hours of do you sleep?**

Behavioral Risk Factor Surveillance System 2014

- 4
- 5
- 6
- 7
- 8
- 9
- 10

**E7.** **Have you smoked at least 100 cigarettes in your entire life?** Behavioral Risk Factor Surveillance System 2014

- No
- Yes 🡪 ( how many **cigarettes**  /day in last month) ----- **cigarettes**  /day

**Thank you for completing this questionnaire**
